# Exercise modulates food reward: neurobiological mechanisms and implications for weight management

**DOI:** 10.3389/fnut.2026.1845848

**Published:** 2026-06-09

**Authors:** Jirui Qiu, Pengfei Ji, Xiaoying Dai, Dan Qiu, Qun Fang, Yansong Li, Tongtong Che, Fanghui Qiu, Shuangshuang Zhang

**Affiliations:** 1 School of Physical Education, Qingdao University, Qingdao, China; 2 Baotou Teachers’ College, Inner Mongolia University of Science and Technology, Baotou, China

**Keywords:** dopamine, exercise, food reward, liking, wanting, μ-opioid receptor

## Abstract

Exercise is widely recommended for body weight management and metabolic health, yet its effects are not explained by energy expenditure alone. Post-exercise food choice, hedonic eating, and compensatory energy intake may attenuate expected training benefits. Food reward, encompassing hedonic “liking” and motivational “wanting,” is therefore a useful framework for understanding variability in exercise-related weight outcomes. For this narrative review, the literature search was performed using titles, abstracts, keywords, and subject headings as search fields. The search strategy was structured around three core concept modules—exercise, food reward, and potential mechanisms—and Boolean operators were used to combine search terms and develop the retrieval syntax. Relevant published studies were identified through EBSCO, ProQuest, PubMed, Scopus, and Web of Science Core Collection from database inception to April 2026. The overall search process was informed by the SANRA (Scale for the Assessment of Narrative Review Articles) principles. The effects of acute and chronic exercise on food reward, together with the neurobiological mechanisms that may contribute to these responses, were examined. Acute exercise may transiently alter the reward value of energy-dense foods, and the direction and magnitude of this effect appear to be influenced by exercise-related factors, including intensity, modality, and time of day, as well as individual factors such as meal-related conditions and body composition. By contrast, chronic exercise may induce adaptive changes in eating behavior and may promote a shift toward healthier dietary patterns. Candidate mechanisms may include changes in mesolimbic dopamine signaling, μ-opioid signaling, insulin-related pathways, glucagon-like peptide-1 signaling, the gut–brain vagal axis, the endocannabinoid system, and stress-sensitive neural circuits. Exercise may also partly counteract diet-induced reward dysfunction and, in some individuals, may be associated with healthier food choice patterns. However, the literature remains heterogeneous because studies differ in population characteristics, exercise protocols, and the reward dimensions assessed. A clearer understanding of these interacting mechanisms may help inform nutrition-informed, exercise-based strategies to support appetite regulation, improve dietary adherence, and enhance long-term weight management.

## 1 Introduction

Insufficient physical activity and excessive energy intake are two major drivers of the global rise in obesity (1, 2). Although exercise is widely regarded as an effective means of maintaining healthy body weight, not all individuals achieve the expected degree of weight loss following exercise interventions. Calories lost during an exercise bout are relatively low compared to daily energy intake, meaning that substantial exercise volumes would be required every day to induce marked body weight loss. Data from the U.S. National Weight Control Registry indicate that some successful weight-loss maintainers expend approximately 728 kcal per week through physical activity yet still derive a relatively high proportion of energy from dietary fat, suggesting that the relationship between physical activity and dietary composition is not linear (3). Because high-fat foods are energy-dense and highly palatable, they often represent a major barrier to energy restriction and weight control in individuals with overweight or obesity (4).

The brain reward system comprises neural structures involved in pleasure, motivation, reinforcement learning, and addictive behavior. Its core pathway is the mesolimbic circuit connecting the ventral tegmental area (VTA) to the nucleus accumbens (NAc) (5). Within this system, food “liking” is more closely related to the opioid system, whereas motivational food “wanting” depends more heavily on dopaminergic signaling. When reward is experienced or anticipated, that is, when hedonic “liking” is engaged, VTA neurons release dopamine into the NAc and prefrontal cortex, thereby reinforcing behavioral motivation (6). Chronic consumption of a high-fat diet may reduce the responsiveness of the reward system to palatable foods, making hedonic satisfaction more difficult to achieve (7). It has therefore been proposed that the positive affect induced by exercise may partially substitute for food-related reward and help regulate post-exercise eating behavior (8).

Energy intake is governed not only by homeostatic mechanisms, but also by hedonic mechanisms, commonly conceptualized as food “wanting” and food “liking,” which together constitute food reward (9). When individuals are exposed to palatable food cues, hypothalamic homeostatic signals interact with reward circuits to shape the motivation to eat (10). Functionally, food reward encompasses both the immediate pleasure derived from sensory properties and the post-ingestive nutrient reward generated by digestive and metabolic signaling; together, these processes influence food preference and subsequent eating behavior (11). Food “liking” is more closely related to endogenous opioid and endocannabinoid systems, whereas food “wanting” depends more heavily on mesolimbic dopamine signaling (12–15). When excessively activated, these reward mechanisms may override homeostatic control, disrupt energy balance, and increase the risk of obesity, binge eating, and other disordered eating behaviors (16). In this review, we summarize the effects of acute and chronic exercise on brain reward circuitry and discuss the major physiological and neural mechanisms that may explain exercise-induced changes in eating behavior, with implications for exercise strategies for body weight management.

From a nutrition and exercise perspective, three questions are especially relevant. First, does exercise reduce the reward value of energy-dense foods or primarily redistribute reward across food categories? Second, which neurobiological pathways help explain interindividual variability in post-exercise food choice and compensatory energy intake? Third, how can these mechanistic insights be translated into exercise prescriptions and dietary strategies that better support appetite control, adherence, and long-term weight management? The following sections address these questions by integrating behavioral and neurobiological evidence, by emphasizing that exercise does not uniformly suppress food reward, and by highlighting the major methodological and phenotypic sources of inconsistency across studies.

## 2 Search strategy

Given that this field encompasses human intervention studies, animal experiments, mechanistic studies, observational research, and different types of reviews, with substantial heterogeneity in study design and outcome measures, a narrative synthesis was considered better suited than quantitative pooling of a single effect size (17). The review process was informed by the principles of SANRA (Scale for the Assessment of Narrative Review Articles), with an explicit description of the search sources, search scope, screening process, and framework for synthesis (18).

The literature search was performed using advanced search functions in EBSCO, ProQuest, PubMed, Scopus, and Web of Science Core Collection. The search period covered studies published from database inception to 20 April 2026. The search strategy was structured around three core concept modules, with terms combined using Boolean operators: (1) exercise, including exercise, training, physical activity, aerobic exercise, resistance exercise, high-intensity interval training, HIIT, acute exercise, and chronic exercise; (2) food reward and related outcomes, including food reward, food preference, food liking, food wanting, implicit wanting, explicit liking, explicit wanting, hedonic eating, food craving, compensatory eating, energy intake, and appetite; and (3) potential mechanisms, including mechanism, dopamine, mesolimbic, reward system, nucleus accumbens, ventral tegmental area, μ-opioid, insulin, GLP-1, PYY, ghrelin, endocannabinoid, vagus, and stress. Search fields varied across databases according to platform-specific functions, the database or platform name, coverage, restrictions, and date of the final search were recorded in a manner broadly consistent with PRISMA-S recommendations, while remaining appropriate to the context of a narrative review (19).

Articles that met the following criteria were screened in: (1) peer-reviewed articles published in English; (2) studies addressing exercise, food reward, or related dimensions such as wanting, liking, food preference, food craving, hedonic eating, compensatory eating, or energy intake; (3) studies reporting the effects of acute or chronic exercise on these outcomes, or examining potential neurobiological or physiological mechanisms relevant to them; and (4) study designs including human intervention studies, observational studies, animal experiments, and narrative reviews, systematic reviews, or meta-analyses directly relevant to the topic of this review. Articles that met the following criteria were screened out: (1) studies not relevant to exercise or food reward; (2) studies focusing only on general appetite, energy metabolism, or appetite-related hormonal changes without addressing food reward, its related behavioral or psychological dimensions, or providing direct or indirect evidence for reward-related mechanisms; (3) conference abstracts, editorials, brief commentaries, dissertations, and informal publications with insufficient methodological information; and (4) non-English publications.

## 3 Effects of acute exercise on food reward

Food reward responses refer to a range of psychophysiological processes through which the brain assigns motivational and hedonic value to food, including craving, pleasure, and learned associations. In this field, the Leeds Food Preference Questionnaire (LFPQ) has been widely used to assess the effects of exercise on food reward. Within this framework, liking generally refers to the subjective hedonic evaluation of food, whereas wanting reflects the motivational drive to approach and obtain food and can be further divided into implicit wanting and explicit wanting (20, 21). Studies reviewed in this section indicate that acute exercise-induced changes in food reward may be influenced by meal-related factors, body composition, exercise characteristics, and the timing of the exercise bout.

### 3.1 Modulatory roles of meal-related factors and body composition in acute exercise-induced food reward responses

Finlayson et al. (22) examined 24 healthy adult women with a mean BMI of 22.3 (± 2.9) kg/m^2^ following 50 min of cycling exercise. Participants whose post-exercise energy intake exceeded exercise-induced energy expenditure were classified as compensators and were compared with a resting control condition. Using a computer-based liking and wanting procedure, explicit liking was assessed with visual analogue scales, relative preference was derived from a forced-choice paradigm based on choice frequency, and implicit wanting was inferred from reaction time. Although no significant between-group difference was observed in explicit liking, participants who exhibited energy compensation showed greater implicit wanting for high-fat foods and a stronger preference for high-fat sweet foods, whereas no such changes were observed in non-compensators. These findings suggest that acute post-exercise food reward responses may be related, at least to some extent, to subsequent total energy intake.

Meal-related factors may also modulate acute exercise-induced changes in food reward. In adolescents with obesity, exercise compared with rest reduced postprandial relative preference for fat and sweetness as well as implicit wanting for high-fat foods (23). However, this modulatory effect may depend on both the type and quantity of nutrients consumed, because when a fixed lunch, rather than ad libitum intake, was provided, no significant differences were observed in subjective appetite or food reward indices, and only choice fat bias differed between pre-meal and post-meal assessments (24). Meal timing may represent an additional source of variability (25). Fillon et al. (26) reported that exercise performed closer to lunch reduced mean energy intake by approximately 170 kcal and lowered both relative and absolute fat intake at dinner. Although preference for high-fat or sweet foods was not significantly reduced, explicit liking for high-fat foods decreased significantly. Meal consumption after exercise also reduced wanting for sweet foods and liking for fat, while increasing liking for sweet foods. By contrast, Miguet et al. (27), in a study using an aquatic exercise intervention, found no significant differences in relative preference, implicit wanting, explicit wanting, or explicit liking for fat and sweet taste dimensions between exercise performed before lunch and exercise performed after lunch. To isolate the specific effect of exercise on food reward more accurately, Yamada et al. (20) and Li et al. (21) therefore suggested that changes in food reward before and after exercise may be better assessed under fasting conditions.

Post-exercise food reward responses may also be related to body composition. In adolescents with obesity and a mean BMI of 35.0 (± 4.3) kg/m^2^, LFPQ scores did not change significantly immediately after a bout of high-intensity interval training (HIIT), whereas ad libitum energy intake decreased significantly, and this reduction was more pronounced in participants with higher BMI and body fat percentage (23). Notably, in a study comparing normal-weight women (22.6 ± 0.93 kg/m^2^) and constitutionally lean women (16.7 ± 1.34 kg/m^2^), both groups received equivalent energy replacement immediately after acute exercise. Under this condition, the constitutionally lean group showed increased explicit liking, explicit wanting, and implicit wanting for sweet relative to savory foods. Although no clear between-group differences were observed in taste-dimension choice, explicit liking, explicit wanting, or implicit wanting, the two groups exhibited different subsequent eating responses. Specifically, ad libitum energy intake was lower than in the resting condition in the constitutionally lean group after exercise with energy replacement, whereas no such change was observed in the normal-weight group (28). The studies summarized above indicate that meal-related factors and body composition may be important moderators of the acute effects of exercise on food reward responses, whereas evidence regarding the role of low BMI remains limited (Table 1).

**Table 1:** Representative studies examining exercise modality, food reward, dietary preference, and related eating outcomes.

Study population (n)	Exercise modality	Intervention protocol	Main findings	Reference
24 healthy women	Acute aerobic exercise	50 min of moderate-to-vigorous exercise on a treadmill or exercise machine (approximately 70% of maximal heart rate)	Compensatory eating after exercise increased, which was associated with enhanced implicit wanting for food and a greater preference for high-fat or sweet foods.	Finlayson et al. (22)
14 healthy young men	Acute aerobic exercise	30 min of running at 70% VO₂max	Explicit and implicit wanting for high-fat foods decreased, whereas preference for sweet foods relative to salty foods increased.	Yamada et al. (20)
16 healthy young men and women	Acute aerobic and resistance exercise	Aerobic exercise: running at 70% VO₂peak; resistance exercise: 12 whole-body exercises performed at approximately 70% 1RM, 12 repetitions per exercise	Preference for high-fat reduced in response to the two exercise modalities; resistance exercise also reduced liking for high-fat foods.	McNeil et al. (29)
33 adolescents with obesity	Acute high-intensity interval training	Five repeated 2 min high-intensity bouts at 70, 75, 80, 85, and 90% maximal heart rate, interspersed with 30 s of low-intensity cycling; total time for 30 min	Preference and implicit wanting for high-energy-dense foods reduced; total energy intake and appetite responses showed downward trends.	Miguet et al. (23)
15 adolescents with obesity	Acute aerobic exercise	30 min of aerobic cycling (65% VO₂peak) for 180 min before lunch, or for 60 min before lunch	60 min before lunch reduced explicit liking for high-fat foods and decreased relative energy intake.	Fillon et al. (26)
12 healthy young men	Acute aerobic exercise	Low intensity at 40% VO₂max and moderate-to-vigorous intensity at 70% VO₂max, for 60 min	Relative preference for sweet foods was higher after high-intensity exercise than after the low-intensity exercise.	Li et al. (21)
17 adolescents with obesity	Acute aerobic cycling	Three crossover conditions: CON, EX-MEAL, and MEAL-EX; 30 min cycling at 65% VO2peak before or after lunch.	Absolute EI was unchanged; relative EI was lower at lunch after both exercise timings, and daily relative EI was lower only in MEAL-EX. Sweet wanting and fat liking were reduced.	Fillon et al. (25)
12 overweight/obese adults	Acute aerobic cycling	Control, MICC, HIIC, and S-HIIC crossover; exercise 1 h after breakfast, ad libitum lunch 3 h after breakfast.	All exercise bouts lowered insulin and increased GLP-1; acylated ghrelin decreased after MICC and HIIC only. EI, appetite, and food reward did not differ.	Martins et al. (32)
19 healthy normal-weight young adults (10 men)	Acute aerobic cycling	CON, HIE, and LIE crossover; 30 min at 75% VO2max or 45 min at 50% VO2max, followed by a fixed lunch.	Appetite, food reward, and later EI did not differ. Exercise reduced some satiety quotient indices, with stronger effects after HIE.	Thivel et al. (30)
45 young Saudi male adults with early or late chronotypes	Acute moderate-intensity cycling	Randomized crossover with a 30-min moderate-intensity cycling bout in the AM or PM after ≥4 h fasting.	Hunger suppression depended on chronotype. AM exercise favored wanting for low-fat sweet foods, whereas PM exercise favored wanting for high-fat sweet and sweet foods.	Beaulieu et al. (33)
10 normal-weight women and 10 women with constitutional thinness	Acute low-intensity cycling with/without post-exercise energy replacement	CON, EX, and EX+R crossover; 30 min cycling at 35% maximal aerobic power, with or without post-exercise energy replacement.	In women with constitutional thinness, EX+R reduced EI vs. CON and increased taste-related reward measures. Hunger was unchanged.	Boscaro et al. (28)
12 adolescents with obesity (9 males)	Acute aquatic exercise	Two-condition crossover: CON or a 45-min aquatic session (~70% peak HR), with ad libitum lunch and dinner.	Lunch and dinner EI, appetite, and food reward did not differ. Daily EI was slightly higher in AQUA, but relative EI was unchanged.	Miguet et al. (27)
20 physically inactive healthy adults	Acute aerobic cycling	Randomized crossover with 40 min cycling at 50% peak power output or 20% peak power output.	Both intensities increased high-fat and savoury wanting, appetite, and cravings. Absolute EI did not differ, but relative EI was lower after moderate-to-vigorous exercise.	Hsieh et al. (31)
13 adolescents with obesity (5 males)	Acute moderate-intensity cycling	Two-condition crossover: CON or 30 min cycling at 65% VO2peak, followed by a fixed lunch and ad libitum dinner.	EI, macronutrient intake, relative EI, and appetite were unchanged. Acute exercise did not alter post-meal appetitive responses.	Siroux et al. (24).
13 sedentary men with obesity and 21 sedentary women with obesity	12-week aerobic training	Cycling at 70% maximal heart rate expending approximately 500 kcal/day, 5 days/week, for twelve weeks	“Non-responders” with smaller reductions in overall body weight/fat showed significant post-exercise increases in food reward (liking and wanting), particularly for high-fat sweet foods, which may attenuate the fat-loss effect of exercise.	Finlayson et al. (36)
2,680 young adults	Aerobic training	Fifteen weeks of aerobic exercise training	Scores for most dietary patterns decreased, with reduced preference for “Western” and “snack” patterns.	Joo et al. (39)
46 adults with overweight or obesity (16 men and 30 women) and 15 non-exercising controls (6 men and 9 women)	12-week aerobic training	Total exercise dose: approximately 10.5 MJ/week	Implicit wanting for high-fat foods decreased, and binge-eating tendency and loss-of-control eating scores were reduced.	Beaulieu et al. (34)
19 men and 39 women with overweight or obesity	Aerobic exercise training	Cycling at 70% VO₂max 5 days/week, expending 500 kcal, for twelve weeks	Controlled eating and inhibition of disinhibition were associated with weight management; participants with a greater tendency toward disinhibition were more likely to achieve successful weight loss during the exercise intervention.	Bryant et al. (38)
49 adult women with overweight or obesity	Moderate-intensity aerobic exercise	Twelve weeks of moderate-intensity aerobic training (EX), targeting 200 min/week, including both supervised and unsupervised sessions, with training content chosen by the exerciser themselves.	Fewer episodes of overeating, internally induced disinhibited eating reduced; stress-induced overeating was not affected.	Unick et al. (35)
32 healthy young adults	High-intensity interval training	Multiple sessions per week; indoor cycling (~70 min/session) with high-intensity bouts and recovery periods.	Spontaneous changes in daily food choice were promoted, with a shift toward a healthier dietary pattern.	Zeppa et al. (41)
304 adolescents with obesity	Aerobic exercise or resistance exercise, or combined exercise	Aerobic training on treadmills, cycle ergometers, and/or elliptical machines (20–45 min/session); resistance training: 3 sets of 7 whole-body exercises; combined training included both components; Total intervention duration: 6 months	External eating and food craving reduced; greater improvements were observed in participants with better adherence after the combined training, and the effects vary according to sex and exercise modality.	Alberga et al. (37)
2,665 Italian adults	Exercise	Based on self-reported exercise habits, with data collected on exercise type, frequency, and average weekly duration, categorized as <5 h, 5–10 h, and >10 h/week.	Physically active individuals preferred healthier foods (plant-based drinks, low-fat yogurt, vegetables, whole grains), with significant sex differences	Campoli et al. (40)

### 3.2 Effects of exercise components on food reward responses

Exercise characteristics themselves may also influence food reward responses. Following isoenergetic aerobic and resistance exercise, healthy young adults showed a significant reduction in relative preference for high-fat foods, but only resistance exercise reduced explicit liking for high-fat foods (29). By contrast, another study reported that isoenergetic high- and low-intensity cycling did not significantly alter any dimension of food reward in healthy normal-weight young adults (30). Evidence from running interventions at different intensities further suggests that high-intensity exercise may increase explicit liking, implicit wanting, and relative preference for sweet foods, while relative preference for high-fat foods remains unchanged (21). This pattern raises the possibility that exercise intensity may influence food reward more clearly along the taste dimension than along the fat-content dimension.

However, findings across the studies discussed above are not fully consistent. Hsieh et al. (31) found that after 40 min of moderate-to-vigorous cycling at 50% peak power output or light cycling at 20% peak power output, participants showed increased implicit wanting for high-fat relative to low-fat foods and for savory relative to sweet foods. Importantly, these changes were observed only for implicit wanting, and no significant differences were found between the two exercise intensities. Similarly, in adults with overweight or obesity, Martins et al. (32) observed that although hormonal responses differed across exercise intensities, hunger, energy intake, and food reward responses did not change significantly.

In addition, the time of day at which exercise is performed may influence food reward responses. Beaulieu et al. (33) reported that 30 min of moderate-intensity cycling performed either in the morning or in the afternoon suppressed hunger in 45 young Saudi men. Morning exercise was associated with greater wanting for low-fat sweet foods, whereas afternoon exercise was associated with greater wanting for high-fat and sweet foods. Furthermore, morning-type participants showed more pronounced hunger suppression after morning exercise, whereas evening-type participants exhibited stronger hunger suppression after afternoon exercise. Across the studies discussed above, the effects of exercise intensity on the fat-related dimension of food reward remain inconsistent. By contrast, selecting an appropriate time of day for exercise may help achieve more favorable food reward responses in some individuals (Table 1).

## 4 Adaptive changes in food reward-related eating behavior and dietary patterns following chronic exercise

Dietary changes following chronic exercise are not limited to reductions in liking, wanting, or relative preference as indices of food reward. Rather, longer-term adaptation may involve broader changes in eating behavior and dietary patterns. Accordingly, many of the studies cited in this section assessed eating-related behavioral or psychological traits to capture the influence of chronic exercise on diet at these two related, but distinct, levels.

### 4.1 Adaptive changes in eating behavior following chronic exercise

Beaulieu et al. (34) emphasized that exercise-induced changes in food reward and eating behaviors should be interpreted separately. In their comparison study, associations between changes in food reward and changes in binge eating or disinhibition were weak. Nevertheless, after 12 weeks of aerobic training, sedentary adults with overweight or obesity showed reduced wanting for high-fat foods as measured by the LFPQ, along with reductions in binge eating and disinhibition as assessed by the Three-Factor Eating Questionnaire (TFEQ) and the Binge Eating Scale. Similarly, a study in women with overweight or obesity who exhibited stress-related eating patterns similarly indicated that 12 weeks of moderate-intensity exercise training reduced the proportion of overeating episodes and decreased disinhibition (35). These findings suggest that chronic exercise may improve eating regulation even when changes in food reward and eating behavior are not tightly coupled.

Evidence from responder analyses further supports this view. When adults with obesity were classified as responders and non-responders according to changes in body composition relative to exercise energy expenditure, fat-loss non-responders showed smaller reductions in fat mass after chronic exercise than fat-loss responders and also exhibited higher overall liking together with greater wanting and relative preference for high-fat sweet foods (36). Similar patterns were observed in analyses derived from the 6-month Healthy Eating, Aerobic and Resistance Training in Youth (HEARTY) randomized controlled trial. Using the Food Craving Inventory, the Dutch Eating Behavior Questionnaire (DEBQ), and the TFEQ to assess food cravings, external eating, emotional eating, restrained eating, and uncontrolled eating, Alberga et al. (37) found that both forms of exercise reduced external eating and food cravings in 304 adolescents with overweight or obesity (BMI 34.6 ± 4.5 kg/m^2^), with the greatest improvements observed in the combined-training group with higher adherence. Although Bryant et al. (38) did not directly assess food reward, they likewise reported that after 12 weeks of supervised exercise, participants with overweight or obesity showed significantly lower disinhibition, higher restraint, and concomitant reductions in body weight and waist circumference. The studies summarized above suggest that chronic exercise may improve behavioral regulatory processes related to overeating and loss-of-control eating, primarily as reflected in changes in eating behavior traits or hedonic eating tendencies (Table 1).

### 4.2 Dietary patterns may shift toward healthier profiles after chronic exercise

Studies examining adaptive changes in dietary patterns following chronic exercise have mainly focused on food choice and overall dietary patterns rather than on direct indices of food reward. At this level, the outcomes most commonly assessed include binge eating, disinhibition, external eating, overeating episodes, and food cravings, whereas relatively few studies have directly measured food reward responses. In a study of 2,680 healthy young adults after 15 weeks of aerobic training, dietary intake across 102 foods was collected using a food frequency questionnaire, and seven dietary patterns were extracted using a Bayesian sparse latent factor model. The results showed that exercise duration was negatively associated with preference for Western and snacking patterns, exercise intensity was positively associated with preference for a prudent pattern, and exercise volume was associated with both lower preference for the snacking pattern and greater preference for the prudent pattern (39). These findings suggest that longer-term exercise exposure may be accompanied by adaptive changes in habitual dietary selection.

Cross-sectional evidence points in a similar direction. Adults who regularly participated in sports were more likely to prefer healthier dietary options, including plant-based beverages, low-fat yogurt, fish, vegetables, fruits, whole grains, tofu, and dark chocolate (40). In addition, participants who completed 9 weeks of progressive HIIT showed a general tendency toward a healthier dietary orientation (41). These studies support the possibility that chronic exercise may facilitate the development of healthier dietary patterns over time (Table 1).

## 5 Dopamine signaling and food reward

**Figure 1:**
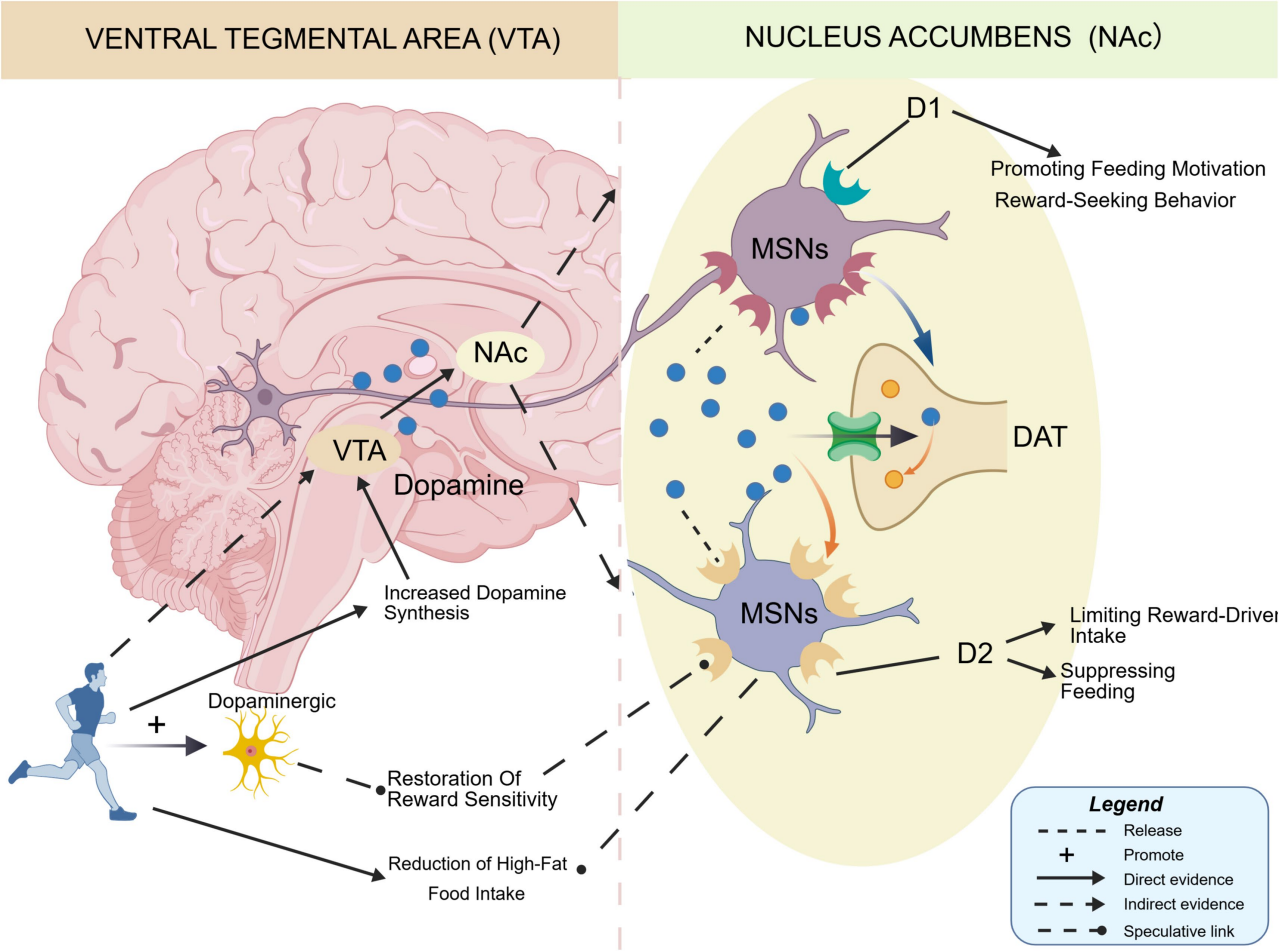
Exercise may modulate the VTA–NAc dopaminergic pathway involved in food reward and high-fat food intake. The dopaminergic projection from the ventral tegmental area (VTA) to the nucleus accumbens (NAc) constitutes a core neural substrate mediating food reward. In the NAc, medium spiny neurons (MSNs) expressing dopamine D1 or D2 receptors represent the primary dopamine-responsive neuronal population. D1 receptor-expressing MSNs in the NAc shell positively regulate feeding behavior, while downregulation of D2 receptors is linked to heightened motivational drive for palatable food. Preclinical animal studies further demonstrate that repeated activation of D1-MSNs combined with inhibition of D2-MSNs promotes food intake and reduces energy expenditure, thereby elevating obesity risk. Conversely, activation of D2-MSNs alongside inhibition of D1-MSNs suppresses feeding, enhances physical activity, and increases energy expenditure. Food-associated sensory cues trigger dopamine release from VTA dopaminergic neurons, and the binding of dopamine to its receptors on NAc neurons amplifies feeding motivation and reward-seeking behavior. Upregulation of the dopamine transporter (DAT) augments synaptic dopamine reuptake, shortens the duration of reward signaling, and may consequently diminish motivation for and preference toward high-fat food. Dietary restriction induces DAT upregulation, whereas voluntary wheel running can partially counteract this adaptive change while providing an alternative reward signal; these changes may contribute to reduced high-fat food consumption. Chronic high-fat diet exposure elicits persistent alterations in feeding behavior that persist even after reintroduction of standard chow; additionally, high-fat feeding reduces sucrose preference, a phenotypic marker of blunted reward system responsiveness. As depicted in this figure, physical exercise increases the number of dopaminergic neurons and dopamine expression in the VTA, and upregulates D2 receptor levels in the NAc in preclinical models. These neuroplastic changes may partially restore reward-related signaling and may contribute to lower high-fat food intake and improved body-weight regulation. Created with BioGDP.com (164).

The dopaminergic pathway from the VTA to the NAc is a key neural substrate of food reward (5). As a key hub of the reward system, the NAc integrates sensory, cognitive, and reward-related information and contributes to goal-directed behavior (42). Dopamine signaling in the NAc is activated by sweet substances, sugars, and corn oil (43–45). Importantly, neural responses to food reward vary across clinical phenotypes: in human studies, individuals with anorexia nervosa often show heightened responsivity to food-related reward cues, whereas individuals with obesity may display relatively blunted responses (46). Studies have shown that moderate-intensity treadmill exercise alters food preference in obese mice by inducing dopaminergic plasticity within the VTA–NAc pathway (47), while scheduled wheel-running access reduces limited-access high-fat food intake through changes in D2 receptor and μ-opioid receptor gene expression in the NAc and VTA (48). The principal neuronal population in the NAc is composed of dopaminoceptive medium spiny neurons (MSNs), which express dopamine D1 and D2 receptors. Animal mechanistic studies have shown that D1 receptor-expressing MSNs in the NAc shell positively regulate feeding (49, 50), whereas downregulation of D2 receptors is associated with heightened motivation for palatable foods (51). Animal studies further suggest that repeated activation of D1-MSNs together with inhibition of D2-MSNs promotes feeding and reduces energy expenditure, thereby increasing obesity risk; conversely, activation of D2-MSNs together with inhibition of D1-MSNs suppresses feeding, promotes activity, and increases energy expenditure (52) (Figure 1).

### 5.1 Exercise may influence food reward by reversing high-fat-diet-induced dopaminergic alterations

Studies in rodents indicate that prolonged exposure to a high-fat diet can produce persistent alterations in feeding behavior, even after standard chow is reintroduced (53). High-fat feeding also reduces sucrose preference, a change closely linked to reduced reward-system responsiveness (54). These findings suggest that long-term consumption of a high-fat diet may impair reward sensitivity and thereby alter subsequent food choice and intake.

Regular exercise has been proposed to modulate dopamine signaling (55, 56) and to partially reverse high-fat-diet-induced abnormalities in reward circuitry and food preference (57). Liang et al. (8) found that intracerebroventricular administration of the μ-opioid receptor agonist DAMGO increased high-fat food intake in rats, whereas in rats with access to voluntary wheel running, the same treatment did not increase total energy intake and was accompanied instead by a reduction in high-fat food intake. This finding raises the possibility that wheel running may attenuate μ-opioid-mediated binge-like responses and may partly reduce motivation for high-fat foods.

Further evidence indicates that, under high-fat-diet conditions, paired-fed rats may undergo dopaminergic adaptations in the VTA–NAc pathway, characterized by reduced D2 receptor expression in both the VTA and the NAc, together with increased expression of the dopamine transporter (DAT) in the VTA (57). Upregulation of DAT enhances the reuptake of dopamine from the synaptic cleft and shortens the duration of reward signaling (58, 59), and may therefore contribute to reduced motivation for and preference toward high-fat foods. At present, however, there is currently no direct evidence supporting the conclusion that “wheel running reduces dependence on high-fat foods through regulation of DAT.” Rather, existing findings suggest that wheel running can directly reduce intake of and preference for a high-fat diet (8), while running training may also be accompanied by increases in the number of dopaminergic neurons and dopamine expression in the VTA, as well as upregulation of D2 receptors in the NAc in obese mice (48). Therefore, the beneficial effect of exercise on high-fat-food-related reward responses should be understood as being associated with remodeling of the VTA–NAc reward pathway, rather than as having been directly demonstrated to be mediated primarily through DAT.

Because food reward and drug reward share substantial overlap within the brain reward system, mechanisms through which exercise attenuates drug addiction may offer indirect insight into how exercise modulates food reward, but they should not be taken as direct evidence for dietary outcomes. Voluntary wheel running changes sensitivity to the rewarding and analgesic effects of morphine. The ventral pallidum (VP), which receives projections from both D1- and D2-MSNs in the NAc, is increasingly recognized as an important node in addiction-related behavior (60, 61). Notably, treadmill exercise and voluntary wheel running may not exert identical effects on reward circuitry (62). Although D1-MSNs and D2-MSNs cannot be reduced to simple “reward” and “aversion” channels, existing evidence suggests that activation of D1-MSNs or inhibition of D2-MSNs in the NAc can facilitate drug reinstatement (63, 64). Moreover, running exercise can reduce enkephalin levels in the VP, restore μ-opioid-related regulation in the VP, reverse the reduction in excitability of NAc D2 receptor-expressing MSNs after morphine withdrawal, and enhance GABAergic inhibition transmitted from D2-MSNs through the VP pathway, thereby persistently suppressing VTA dopamine neurons (65). These findings imply that exercise may indirectly influence food reward by reshaping the functional properties of the NAc–VP–VTA circuit.

Food-related cues promote dopamine release from VTA dopaminergic neurons, and dopamine binding to receptors on NAc neurons enhances food-seeking motivation (66). By contrast, DAT clears dopamine from the synaptic cleft and therefore restrains dopamine-mediated food reward signaling (67). Loss of D2 receptor signaling in the NAc has been closely associated with binge-like eating (51). When D2 receptor levels are downregulated, dopamine transmission along the VTA–NAc pathway is weakened; as a result, individuals with obesity may experience blunted postprandial reward signals and may require greater food intake to achieve sufficient reward (68). In obese mice, 8 weeks of running increased the number of dopaminergic neurons and dopamine expression in the VTA and upregulated D2 receptor levels in the NAc. These changes are consistent with the possibility that exercise may partially restore reward-related signaling in this animal model and may be associated with reduced high-fat food intake and improved body-weight control in obese mice (48). Notably, this study did not observe a marked change in DAT expression, suggesting that the beneficial effect of exercise on food reward is not fully dependent on DAT-mediated mechanisms. These preclinical findings may help explain how exercise may influence reward-related responses to palatable food, but whether such circuit-level adaptations translate into healthier food choices in humans remains uncertain (Figure 1).

### 5.2 Exercise may influence food reward by modulating insulin signaling within dopamine circuits

Insulin receptors are expressed on VTA dopaminergic neurons. After feeding, insulin enters the circulation, crosses the blood–brain barrier, and acts on VTA dopaminergic neurons to reduce neuronal excitability, thereby decreasing dopamine release into target regions such as the NAc (69). Mouse studies further suggest that this insulin-mediated reduction in food reward depends to a large extent on DAT: when DAT is pharmacologically blocked, or when VTA tissue from DAT knockout mice is examined, the inhibitory effect of insulin on dopamine release is abolished (70). Chen et al. (71) reported that 8 weeks of aerobic exercise improved insulin sensitivity and reduced NAc dopamine levels during feeding in obese rats. These changes were interpreted as being consistent with ameliorated obesity-induced reward-related dysregulation and were accompanied by lower fat preference, reduced excessive high-fat diet intake, slower body weight gain, and improved body composition in this model.

**Figure 2:**
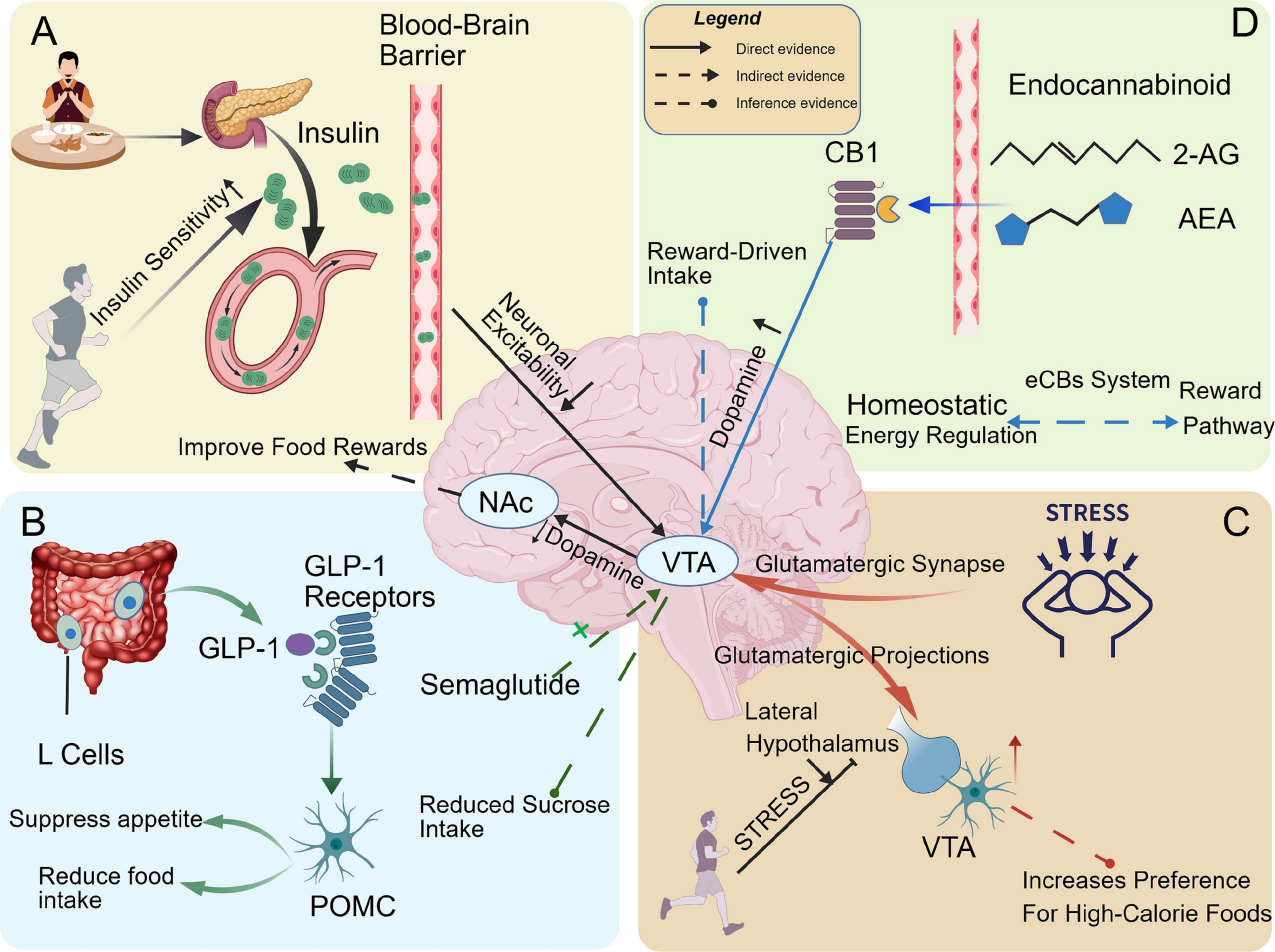
Candidate peripheral metabolic and stress-related signals converge on the mesolimbic dopaminergic system to regulate food reward and food intake. **(A)** After food intake, insulin enters the circulation, crosses the blood–brain barrier, and acts on dopaminergic neurons in the ventral tegmental area (VTA) to reduce their excitability, thereby decreasing dopamine release to target regions such as the nucleus accumbens (NAc). Aerobic exercise improves insulin sensitivity in obese rats and lowers dopamine levels in the NAc during feeding; these findings may partly reflect reduced reward-related dysregulation and were accompanied in this model by lower fat preference, reduced excessive high-fat diet intake, and improved metabolic outcomes. **(B)** Glucagon-like peptide-1 (GLP-1), secreted by L cells in the ileum and colon in response to dietary fat and protein, activates GLP-1 receptors (GLP-1R) and increases proopiomelanocortin (POMC) neuronal activity, thereby enhancing satiety, suppressing appetite, and reducing food intake. High-dose semaglutide may also reduce sucrose intake by enhancing the activity of VTA dopaminergic neurons. **(C)** Peripheral endocannabinoids, including 2-arachidonoylglycerol (2-AG) and anandamide (AEA), can cross the blood–brain barrier and bind to cannabinoid receptor 1 (CB1), thereby promoting dopamine release in reward-related regions such as the NAc. The endocannabinoid system may therefore serve as a key bridge between homeostatic energy regulation and hedonic reward pathways. **(D)** Stress enhances glutamatergic synaptic transmission onto VTA dopaminergic neurons. Social stress activates glutamatergic neurons in the lateral hypothalamus that project to the VTA and strengthens their signaling to dopaminergic neurons through AMPA receptor-related mechanisms, thereby increasing dopamine signaling and preference for high-calorie foods. Exercise is commonly used as a strategy for stress reduction. Together, these pathways illustrate candidate routes through which insulin, GLP-1 signaling, endocannabinoids, and stress-related glutamatergic inputs may converge on the VTA–NAc reward circuit to influence food reward, appetite, and dietary choice. Created with BioGDP.com (164).

A small population of cholinergic interneurons in the NAc also expresses high levels of insulin receptors. Insulin activates the PI3K–Akt pathway in these neurons and thereby promotes cholinergic activity (72, 73). Acetylcholine released from these neurons can act on nicotinic acetylcholine receptors located on neighboring dopamine terminals, directly evoking dopamine release or enhancing terminal responsiveness to action potentials originating from VTA cell bodies (74, 75). Thus, insulin may exert bidirectional and highly localized control over food reward signaling by acting at different levels of the reward circuit. Glycogen synthase kinase 3β (GSK-3β), a serine/threonine kinase, can attenuate D1 receptor-mediated excitatory signaling through phosphorylation of downstream proteins such as DARPP-32 (72). When GSK-3β itself is phosphorylated, its activity is reduced, potentially weakening the inhibitory tone imposed on reward processing (76, 77). High-fat-diet-induced obesity has been shown to impair insulin signaling in the NAc and blunt insulin-dependent dopaminergic regulation, whereas aerobic exercise may partly reverse these alterations by increasing insulin receptor expression, phosphorylated Akt, and phosphorylated GSK-3β (71, 72, 78). Such changes may contribute to partial restoration of reward-related signaling and are consistent with the possibility that eating behavior may also improve in this model (71) (Figure 2).

### 5.3 Additional factors that modulate dopamine signaling

#### 5.3.1 Gut peptides

##### 5.3.1.1 Glucagon-like peptide-1

Glucagon-like peptide-1 (GLP-1) is secreted by L cells distributed throughout the small intestine and colon in response to dietary lipids, proteins, and sugars. In addition to this peripheral source, GLP-1 is also produced within the central nervous system, particularly by preproglucagon neurons located in the hindbrain nucleus tractus solitarius (79, 80). Centrally produced GLP-1 can act through activation of the GLP-1 receptor (GLP-1R), and glucagon-like peptide-1 receptor signaling engages both homeostatic and reward-related brain regions, including brainstem nuclei such as the area postrema and nucleus tractus solitarius, hypothalamic sites such as the arcuate nucleus and paraventricular nucleus, and mesolimbic structures such as the ventral tegmental area and nucleus accumbens, thereby supporting a role for glucagon-like peptide 1 in integrating satiation, metabolic control, and food reward (81). Accordingly, the central GLP-1 system is anatomically and functionally positioned to influence satiation, metabolic regulation, food reward, and stress-related feeding responses (80, 82). It increases proopiomelanocortin (POMC) neuronal activity, enhances satiety, suppresses appetite, and reduces food intake (83). GLP-1 also prolongs satiety by delaying gastric emptying (84).

In human intervention studies, semaglutide, a GLP-1R agonist, has shown substantial efficacy in the treatment of obesity (85) and has been reported to reduce food craving as well as preference for high-fat foods in individuals with obesity (86, 87). These effects may involve the nucleus tractus solitarius, arcuate nucleus, and lateral septal nucleus, all of which are implicated in dopamine transmission and reward-related behavior (88, 89). Beyond pharmacological intervention, exercise, as a non-pharmacological strategy capable of modulating incretin responses, may also participate in the regulation of appetite and food reward by influencing GLP-1 levels. Howe et al. (90) reported that both moderate-intensity exercise at 65% VO₂max and high-intensity exercise at 85% VO₂max increased GLP-1 levels and suppressed appetite in trained female athletes. Similarly, in healthy men, GLP-1 levels increased significantly after high-intensity aerobic interval exercise and were positively associated with reductions in appetite (91). A systematic review and meta-analysis further showed that both acute exercise at 55–65% of maximal heart rate and chronic exercise at 65–85% of maximal heart rate significantly increased GLP-1 levels in individuals with type 2 diabetes (92). The human studies and meta-analysis cited above suggest that exercise may influence food reward, at least in part, through modulation of GLP-1 levels. Additional rodent studies indicate that semaglutide can reduce intake of high-calorie foods such as chocolate (93). Animal experiments further suggest that high-dose semaglutide may reduce sucrose intake by enhancing the activity of VTA dopaminergic neurons, suggesting that GLP-1R agonism may alter food intake partly through changes in reward-system sensitivity. However, high-dose GLP-1R agonists, including semaglutide, may induce nausea and related discomfort (94, 95), whereas low-dose semaglutide may have limited effects on food motivation (96). Whether exercise combined with low-dose semaglutide could optimize food reward regulation while minimizing adverse effects warrants further investigation (Figure 2).

##### 5.3.1.2 Ghrelin

Ghrelin, also known as the hunger hormone, is an acutely regulated peptide hormone produced primarily by X/A-like cells in the gastric fundus. Acylated ghrelin, which is generated through the catalytic action of ghrelin O-acyltransferase (GOAT), is currently the only gut-derived orexigenic hormone that has been definitively identified. By binding to growth hormone secretagogue receptor type 1a (GHSR-1a) expressed on vagal afferents (97) and within hypothalamic nuclei (98), ghrelin activates AgRP neurons and indirectly inhibits POMC neuronal activity, thereby promoting appetite. In addition to its role in homeostatic appetite regulation, ghrelin has also been implicated in reward-related processes, particularly the mesolimbic dopamine system. A meta-analysis indicated that chronic exercise may increase ghrelin levels in individuals with overweight or obesity (99).

Animal evidence further suggests that ghrelin may modulate food reward through dopaminergic mechanisms. Engel et al. (100) reported that systemic administration of ghrelin enhanced locomotor stimulation, dopamine release in the nucleus accumbens shell, and conditioned place preference. These effects were attenuated by inhibition of nitric oxide synthase or by local blockade of soluble guanylate cyclase within the ventral tegmental area (VTA). *In vivo* electrochemical recordings further showed that ghrelin increased nitric oxide levels in the VTA, suggesting that ghrelin may, at least in part, facilitate mesolimbic dopamine signaling through a VTA nitric oxide–cGMP pathway. In addition, Edwards et al. (101) showed in animal studies that the facilitative effect of ghrelin on food motivation partly depends on the endocannabinoid system within the VTA. Blockade of CB1 receptors attenuated ghrelin-induced food-seeking behavior as well as the enhancement of excitatory input onto VTA dopamine neurons, whereas inhibition of endocannabinoid degradation amplified these effects. These findings raise the possibility that ghrelin may enhance dopamine output related to food motivation by recruiting endocannabinoid signaling.

##### 5.3.1.3 Peptide YY

Peptide YY (PYY) is secreted mainly by L cells in the ileum and colon (102). It circulates in two principal forms: the full-length 36-amino-acid peptide PYY1–36 and the truncated 34-amino-acid peptide PYY3–36. PYY3–36 is the predominant circulating form and exerts a stronger anorexigenic effect (103). As one factor capable of modulating PYY levels, exercise has been shown to significantly increase total PYY and PYY3–36 concentrations following sprint interval exercise, endurance exercise, and resistance exercise (90, 104–106). Beyond its role in appetite suppression, PYY may also regulate food intake by acting on brain regions involved in food reward and learning. Human functional magnetic resonance imaging (fMRI) studies have shown that administration of PYY3–36 activates neurons within the mesocorticolimbic dopamine pathway (107), and animal studies have further demonstrated that exogenous PYY3–36 increases dopamine synthesis and release in the rat striatum (108). Stadlbauer et al. (109) further found that peripheral injection of PYY3–36 enhanced behavioral responses to novelty and to dopaminergic drug challenge in mice. However, PYY3–36 did not directly activate dopaminergic neurons in the VTA or substantia nigra, but instead significantly activated GABAergic cells, suggesting that PYY3–36 may be more likely to influence dopamine function indirectly through striatal GABAergic pathways.

#### 5.3.2 The gut–brain vagal pathway and the endocannabinoid system

The gut–brain vagal pathway is another important pathway involved in reward-related eating (110, 111). Peripheral endocannabinoids (eCBs) participate in feeding regulation and metabolic efficiency and may influence reward-driven intake (112, 113). In humans and other mammals, 2-arachidonoylglycerol (2-AG) and anandamide (AEA) are the two major endocannabinoids. Both can cross the blood–brain barrier and bind to CB1 receptors, thereby promoting dopamine release in reward-related regions such as the NAc (114). Endocannabinoid signaling in the NAc and VTA modulates dopamine release associated with hedonic feeding (115, 116). During hunger, hypothalamic endocannabinoid levels rise, whereas endocannabinoid levels in the NAc decline during food consumption. Accordingly, the endocannabinoid system may serve as a key bridge between homeostatic energy regulation and hedonic reward pathways; its principal receptors are the G-protein-coupled receptors CB1 and CB2 (117). Studies have shown that high-sugar intake increases CB2 receptor mRNA in the NAc, whereas CB1 receptor mRNA is reduced in obesity-prone rats (118). Binge-like eating may also induce a compensatory adaptation that depends on the gut–brain axis, being mediated through the vagus nerve and dependent on peripheral eCB signaling. Selective inhibition of peripheral CB1 receptors can enhance vagal-dependent hypothalamic activity, alter metabolic efficiency, dampen mesolimbic dopamine circuit activity, and ultimately suppress intake of palatable food (119) (Figure 2).

#### 5.3.3 Stress

Stress may increase the intake of calorie-dense foods and thereby contribute to obesity (120, 121). Stress-induced eating likely involves alterations in reward-system function (122), in which VTA dopamine neurons play a key role in food reward and motivation (123, 124). Both human and animal studies indicate that chronic stress can disrupt the balance between homeostatic and hedonic control of eating. For example, repeated stress has been shown to increase fat preference in mouse studies (125), although not all studies have found a significant effect of chronic stress on preference for highly rewarding foods (126). In rodents, stress enhances glutamatergic synaptic transmission onto VTA dopamine neurons (127). The lateral hypothalamus is also critically involved in the control of palatable food intake (128, 129). Functional magnetic resonance imaging studies have shown that resting-state connectivity between the lateral hypothalamus and midbrain is positively associated with emotional eating tendencies in individuals with overweight (130). Further work in rodent models indicates that social stress activates glutamatergic lateral hypothalamic neurons projecting to the VTA and strengthens their signaling to dopamine neurons via AMPA receptor-related mechanisms, while also increasing lateral hypothalamic regulation of dopamine output toward key targets including the prefrontal cortex (131).

Exercise is often used as a strategy for stress reduction (132), and some studies suggest that it may modulate preference for high-fat foods (36). However, one human study reported that following a single bout of acute aerobic treadmill running performed at approximately 70% VO₂peak prior to exposure to an acute psychological stressor induced by the Trier Social Stress Test, circulating ghrelin concentrations were lower after exercise, whereas neither total energy intake nor intake of unhealthy foods differed significantly from the control condition (133). Thus, it remains unclear whether exercise can fully reverse stress-induced circuit-level changes that promote excessive intake of palatable foods, or whether exercise can reliably improve stress-related disturbances in food reward (Figure 2).

## 6 μ-Opioid signaling and food reward

The neural network underlying the hedonic “liking” component of reward includes the brainstem, pons, nucleus accumbens, ventral pallidum, amygdala, and taste-related pathways within the prefrontal cortex (134, 135). μ-Opioid receptor signaling in the NAc, ventral pallidum, and related regions enhances the “liking” of natural rewards such as palatable foods, as well as that of drugs of abuse (136). Increased food intake induced by μ-opioid receptor agonists is generally associated with enhanced food reward and hedonic valuation (137, 138). Endogenous opioid peptides and their receptors are also widely expressed in homeostatic feeding centers such as the hypothalamus, including β-endorphin, enkephalin, dynorphin, and their corresponding receptors. Administration of the μ-opioid receptor agonist DAMGO into the NAc preferentially increases intake of high-fat diets (139). A large body of evidence indicates that the opioid system promotes reward-driven eating (140, 141): mice lacking μ-opioid receptors or β-endorphin show reduced binge-like eating and diminished food reward, whereas mice lacking enkephalin exhibit less marked changes, suggesting a more prominent role for μ-opioid receptors and β-endorphin in promoting food reward (142).

**Figure 3:**
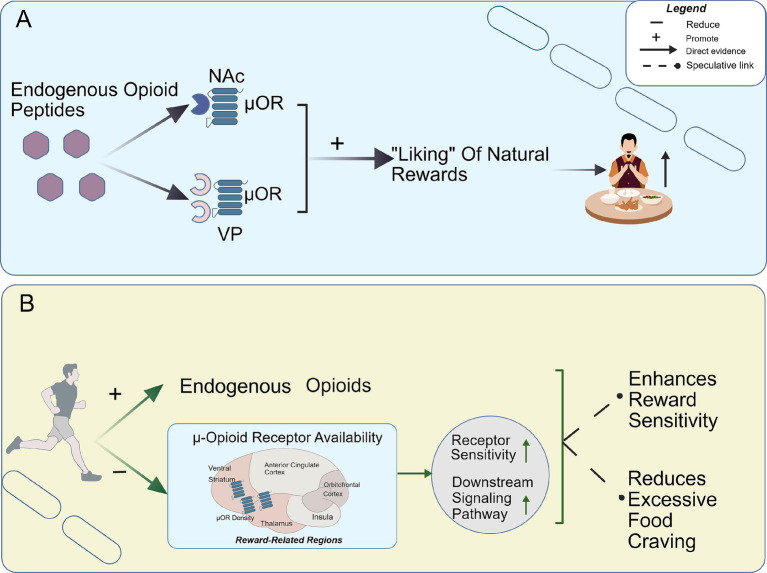
Exercise may modulate μ-opioid receptor signaling in reward-related regions involved in hedonic food reward and high-fat food intake. μ-Opioid receptor signaling in reward circuits and its modulation by exercise. **(A)** In the nucleus accumbens (NAc), ventral pallidum (VP), and related reward-associated regions, endogenous opioid peptides activate μ-opioid receptors (μORs) to enhance the hedonic “liking” of natural rewards, including palatable foods. **(B)** Exercise may modulate this system by promoting endogenous opioid release and reducing μOR availability in reward-related regions, including the thalamus, anterior cingulate cortex, orbitofrontal cortex, and insular cortex. Notably, a greater post-exercise reduction in μOR binding potential is associated with a greater increase in anticipatory food reward responses. Reduced receptor availability may induce compensatory cellular adaptations, possibly reflected by enhanced receptor sensitivity and/or changes in receptor-coupled signaling pathways, although these mechanisms have not yet been fully elucidated. Within the framework illustrated here, exercise-induced opioid release, together with adaptive changes in μOR function, may contribute to altered reward sensitivity and may be consistent with reduced excessive food craving.Created with BioGDP.com (164).

By contrast, hypothalamic POMC neurons and prodynorphin neurons are generally associated with suppression of food intake (143, 144). Nevertheless, even in the sated state, sugar intake can engage μ-opioid-related signaling to maintain motivation for sugar consumption and promote overeating (145). Opioid signaling also interacts with hunger circuits; for example, *κ*-opioid signaling on AgRP neurons suppresses intake of palatable foods and reduces AgRP neuronal excitability (146). Following consumption of palatable food, β-endorphin levels in cerebrospinal fluid and blood rise, and μ-opioid receptor expression in the mesolimbic system may also increase (147–149). In a human imaging study, even intake of non-palatable food may increase opioid activity; a human study reported increased forebrain μ-opioid receptor availability after ingestion of non-palatable food, suggesting that the endogenous opioid system also participates in post-ingestive reward processing (150). Because opioid signaling appears to peak approximately 10 min after feeding, digestion itself may be an important trigger of opioid release. Notably, AgRP neurons also express D2 dopamine receptors, and dopamine binding inhibits AgRP neuronal excitability. β-Arrestin, an intracellular signaling protein, can mediate μ-opioid receptor desensitization; downstream pathways, particularly β-arrestin/PI3K-mediated μ-opioid signaling, may rapidly influence AgRP membrane potential and reduce food intake (151) (Figure 3).

### 6.1 Exercise may influence food reward by modulating μ-opioid receptor signaling

The μ-opioid receptor system can elicit pleasurable and satisfying hedonic experiences and is central to the regulation of palatable food intake and reward processing (152, 153). In human neuroimaging studies, Saanijoki et al. (154) found that the greater the reduction in μ-opioid receptor binding potential after aerobic exercise, the greater the increase in anticipated food reward responses in reward-related regions such as the ventral striatum, medial prefrontal cortex, anterior cingulate cortex, and insula. This observation is consistent with the possibility that exercise may increase endogenous opioid release and alter reward-system responsiveness, which may partly enhance sensitivity to non-food rewards. In another human study, high-intensity interval exercise induced endogenous opioid release and significantly decreased μ-opioid receptor availability in the thalamus, anterior cingulate cortex, orbitofrontal cortex, and insular cortex, whereas moderate-intensity aerobic exercise did not produce a comparable pattern (155). A post-exercise reduction in μ-opioid receptor availability is generally interpreted as reflecting increased endogenous opioid release and receptor occupancy. In parallel, β-endorphin levels often increase after high-intensity or prolonged exercise, whereas comparable changes are not consistently observed after low- or moderate-intensity exercise (156), suggesting that opioid-like reward responses differ across exercise intensities.

In humans, habitually active individuals may show greater exercise-induced brain opioid release after high-intensity interval training, and their hedonic experience may more readily reach a state of satisfaction. Moreover, physically active individuals exhibit larger post-exercise reductions in μ-opioid receptor binding in the anterior cingulate cortex, insula, orbitofrontal cortex, and ventral striatum, which may partly relate to reductions in eating motivation or food craving (157).

The lateral hypothalamus contributes to reward processing (158), and μ-opioid receptor-expressing neurons in this region can influence energy intake (159, 160). Animal work has shown that wheel running reduces the choice of high-fat/high-sugar diets and alters voluntary dietary selection in a time-dependent manner, although no clear changes in the expression of genes related to opioid or dopamine signaling were detected in the lateral hypothalamus or NAc in that study (161). Additional studies using intracerebroventricular administration of μ-opioid receptor ligands further suggest that repeated exposure to exercise reward may induce adaptive changes in opioid components of the reward pathway (8). ΔFosB, a highly stable transcription factor, can drive persistent plastic remodeling of reward circuitry through long-term regulation of downstream target genes (162). Exposure to addictive drugs increases ΔFosB levels in both the core and shell of the NAc (163), and six weeks of voluntary wheel running similarly increases FosB/ΔFosB expression in these regions while upregulating selected opioid receptor mRNAs (56). These findings support the possibility that exercise reshapes the μ-opioid system and related transcriptional programs, enhances non-food reward, and may partly substitute for the hedonic reinforcement derived from palatable foods.

Within the framework discussed in this section, the μ-opioid system primarily maps onto the “liking” dimension of food reward, and exercise may influence this dimension by altering endogenous opioid-like reward responses. However, the extent to which exercise-induced changes in μ-opioid signaling reduce reliance on highly palatable foods remains indirect, particularly when extrapolating from animal studies or receptor-availability findings to human eating behavior. Accordingly, the relevance of this framework to nutrition-focused exercise interventions should be interpreted cautiously (Figure 3).

## 7 Conclusion

Across the human and animal studies discussed in this review, exercise may influence food reward through multiple interacting pathways, including appetite-regulating hormones, insulin, GLP-1, ghrelin, and PYY that affect dopamine-related food wanting, as well as possible changes in μ-opioid signaling involved in hedonic food liking. However, because the mechanisms underlying food reward are highly complex and because exercise-induced changes in food reward are shaped by individual characteristics such as body composition and behavioral phenotype, the available findings remain heterogeneous. Accordingly, an important challenge for future research is to better account for the influence of non-target factors, including mechanistic and individual-difference-related variables, on observed outcomes.

From an applied perspective, several practical issues may warrant consideration when designing exercise prescriptions for weight management in combination with nutritional intervention. In individuals prone to compensatory eating after exercise, particular attention should be paid to structured post-exercise meal planning rather than unrestricted ad libitum eating. When an individual’s food reward response appears to be sensitive to exercise intensity, higher-intensity exercise might be more effective for weight control. Exercise performed closer to a meal may in some cases be more likely to suppress certain dimensions of food reward.

In addition, chronic exercise may be especially important because it may not only help attenuate food reward responses, but also support healthier eating behaviors and dietary patterns over time. Therefore, for individuals seeking weight management or obesity prevention, sustained participation in exercise may be helpful in achieving meaningful benefits through the combined effects of increased energy expenditure and possible adaptive changes in dietary behavior.
